# High Triplet Energy Host Materials for Blue TADF OLEDs—A Tool Box Approach

**DOI:** 10.3389/fchem.2020.00657

**Published:** 2020-07-29

**Authors:** Francesco Rodella, Sergey Bagnich, Eimantas Duda, Tobias Meier, Julian Kahle, Stavros Athanasopoulos, Anna Köhler, Peter Strohriegl

**Affiliations:** ^1^Macromolecular Chemistry I, University of Bayreuth, Bayreuth, Germany; ^2^Soft Matter Optoelectronics, University of Bayreuth, Bayreuth, Germany; ^3^Departamento de Física, Universidad Carlos III de Madrid, Madrid, Spain; ^4^Bayreuth Institute of Macromolecular Research (BIMF), University of Bayreuth, Bayreuth, Germany

**Keywords:** thermally activated delayed fluorescence, TADF, host, organic light emitting diode, tool box approach, high triplet energy

## Abstract

The synthesis of stable blue TADF emitters and the corresponding matrix materials is one of the biggest challenges in the development of novel OLED materials. We present six bipolar host materials based on triazine as an acceptor and two types of donors, namely, carbazole, and acridine. Using a tool box approach, the chemical structure of the materials is changed in a systematic way. Both the carbazole and acridine donor are connected to the triazine acceptor via a para- or a meta-linked phenyl ring or are linked directly to each other. The photophysics of the materials has been investigated in detail by absorption-, fluorescence-, and phosphorescence spectroscopy in solution. In addition, a number of DFT calculations have been made which result in a deeper understanding of the photophysics. The presence of a phenyl bridge between donor and acceptor cores leads to a considerable decrease of the triplet energy due to extension of the overlap electron and hole orbitals over the triazine-phenyl core of the molecule. This decrease is more pronounced for the para-phenylene than for the meta-phenylene linker. Only direct connection of the donor group to the triazine core provides a high energy of the triplet state of 2.97 eV for the carbazole derivative **CTRZ** and 3.07 eV for the acridine **ATRZ**. This is a major requirement for the use of the materials as a host for blue TADF emitters.

## Introduction

Organic light emitting diodes (OLEDs) are overtaking the field of display applications due to several favorable characteristics in comparison with classic liquid crystal devices (LCDs) such as lower energy consumption and simpler technology (Endo et al., 2011; Sasabe and Kido, 2013). For these reasons, in the past years, much effort was dedicated to develop new materials and technologies for OLEDs. The current focus is, in particular, to improve their efficiency and long-time stability. Starting from classic fluorescent emitters (1st generation) with an internal quantum efficiency (IQE) limited to 25%, research moved to phosphorescence emitters (2nd generation) with an increased IQE of 100% and then to thermally activated delayed fluorescence (TADF), which represents the last and very promising generation of OLED materials (Endo et al., 2011; Uoyama et al., 2012). In comparison with phosphorescence emitters that use less available and more expensive noble metals, TADF emitters often rely on pure organic molecules with donor and acceptor groups. This donor-acceptor structure often leads to a small singlet (S_1_)-triplet (T_1_) energy gap (ΔE_ST_) that allows reverse intersystem crossing (RISC) from the triplet into the singlet state, permitting a theoretical 100% IQE by emission of prompt and delayed fluorescence. Lin et al. reported a state of the art TADF OLED with an IQE of 100%, and, more surprisingly, an external quantum efficiency (EQE) of 37%. So far this is one of the highest efficiency ever reported for TADF emitters (Lin et al., 2016). Another promising way to obtain TADF molecules with pure emission is based on multiple resonance effect (Hatakeyama et al., 2016). Most of the work on TADF focuses on new emitters. Pure emitter layers usually show self quenching, thus it is necessary to dilute the emitters in suitable host materials. These hosts have to fulfill several requirements such as having a high triplet energy level, chemical and thermal robustness, and balanced charge carrier mobilities (Wong and Zysman-Colman, 2017). One of the recent challenges is to develop efficient and stable blue OLED host/emitter systems that can replace the less efficient fluorescent ones of the 1st generation, currently used in display applications (Cai and Su, 2018). While designing a host, the triplet energy level is the first key point to look at, especially if the aim is to use it for blue emitters since they require hosts with a triplet energy of about 3 eV. So far, the reported hosts with a triplet energy suitable for blue emitters are mostly monopolar and made with groups such as phosphine oxide, which are known to have limited stability (Hirata et al., 2014; Chen et al., 2015; Kim et al., 2015; Yook and Lee, 2015; Zhang et al., 2015; Chatterjee and Wong, 2018). Among them, DPEPO is one of the most used hosts for TADF blue emitters since it has a high energy triplet level of 3 eV (Han et al., 2011). Several carbazole-based hosts have been developed, with the advantage to have higher stability. Some of them, still monopolar, come from the 2nd generation such as mCP and mCBP (Wong and Zysman-Colman, 2017). Successively, in order to have a balanced charge carrier transport, bipolar carbazole-based hosts were developed by adding acceptor units such as cyano, triazine, and phosphine oxide groups (An et al., 2011; Ding et al., 2015; Kukhta et al., 2017; Shin et al., 2018) or formation of dimers and trimers (Tomkeviciene et al., 2011). Most of these carbazole-based hosts are limited to the use of blue greenish emitters since their triplet energies are below 3 eV. Hosts with less common functional groups such as cyclophosphazene (Nishimoto et al., 2014), silicon (Ren et al., 2004), and benzimidazobenzothiazole (Cui et al., 2016a) were also developed in order to increase the triplet energy. Furthermore, trying to avoid the problem of suitable hosts, non-doped emitting layers are also under investigation by using sterically demanding molecules such as dendrimers, but they still suffer from lower efficiencies in comparison with doped emitting layers (Wong and Zysman-Colman, 2017; Cai and Su, 2018; Wei et al., 2018). Overall, host materials remain less developed in comparison with emitters, therefore some standard approaches and guidelines could be helpful to progress this field.

**Figure 1:**
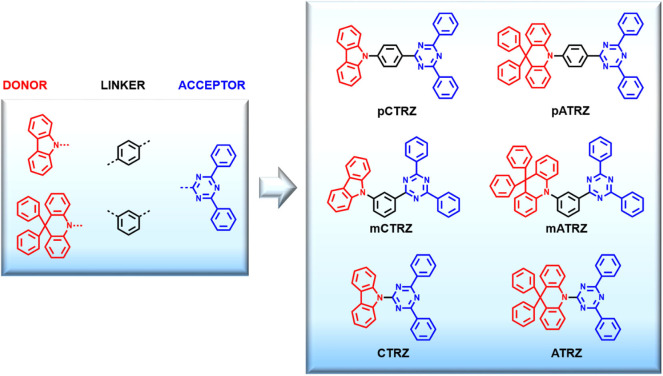
Tool box approach. Chemical structures of the molecular constituents used and the resulting target compounds, along with their names.

In this paper we present a systematic study called “tool box approach” illustrated in Figure 1 in order to understand how to increase the triplet energy of triazine-based bipolar molecules. Namely, one acceptor (triazine), two linkers (meta and ortho phenyl rings), and two donors (carbazole and acridine) were chosen as building blocks because of their stability and common use, then combined to obtain six molecules (**pCTRZ**, **mCTRZ**, **CTRZ**, **pATRZ**, **mATRZ**, **ATRZ**), of which two are new (**mATRZ** and **ATRZ**). All the target compounds were compared through spectroscopic characterization and theoretical calculations. This allows to understand the contribution of the single building blocks on the triplet energy of the final molecules and so to give a structure-property relationship. We note that spectroscopic and computational data on **pCTRZ**, **mCTRZ**, **CTRZ**, **pATRZ** are already available, yet sometimes not complete across the series, and sometimes contradictory. Here we study the entire set of 6 compounds under identical conditions which facilitates comparison. We show that by using a direct linkage between donor and acceptor it is possible to reach triplet T_1_ energy values above 3 eV. The strategy here proposed can be helpful to design new bipolar molecules with high triplet energies and stable functional groups, permitting to obtain potential hosts for blue TADF OLEDs.

## Methods

### Materials

All the starting materials needed for the synthesis were purchased from abcr and Acros Organics. The two reference compounds **pATRZ** and **pCTRZ** were purchased from Lumtec. The reference compounds CBP and spiro-2CBP were purchased from Sigma Aldrich. mCBP was synthesized as described in literature (Schroegel et al., 2011). Their chemical structures can be found in the Supplementary Information.

### Purification

The synthesized compounds **mCTRZ**, **mATRZ**, **CTRZ**, and **ATRZ** were purified by train sublimation in a Carbolite split tube furnace HZS 12/450.

### Characterization

^1^H NMR and ^13^C NMR spectra were recorded on a Bruker Avance III HD (500 MHz) and the chemical shifts were referred to chloroform-d3 (7.26 ppm). MS spectra were obtained on a Finningan MAT 8500 using electron impact ionization.

### Photophysical Measurements

For the absorption and emission measurements, we prepared solutions of 0.05 mg/ml of the compounds in toluene. Absorption spectra were recorded at room temperature on a Cary 5000 double beam spectrophotometer. Steady-state and time resolved photoluminescence spectra in solution and films were recorded at 298 and 77 K using a Jasco FP-8600 spectrofluorometer. For **pCTRZ** and **pATRZ**, fluorescence could be taken at 77 K in steady state operation without superimposed phosphorescence signal. For **mCTRZ** and **mATRZ**, the stronger intensity of the phosphorescence required us to use the chopped excitation from the Jasco spectrofluorometer and gated detection (delay time of 50 μs, integration time of 50 μs) in order to obtain only fluorescence without contribution of the phosphorescence. To exclude the even stronger phosphorescence signal in the fluorescence measurements of **CTRZ** and **ATRZ**, we used a 355 nm pulsed laser excitation with iCCD camera, a delay time of 10 ns and an integration time of 30 ns.

Values for the photoluminescence quantum yield (PL QY) of the molecules in solution were obtained using the Jasco FP-8600 spectrofluorometer equipped with an integrating sphere. The intensity decay of the fluorescence was measured using a Q-switched QS laser MPL15100-DP at λ = 355 nm as the excitation source and an Andor iCCD camera (iStar A-DH334T-18F-03) as the detector. Rate constants for the decay of singlet state were calculated using following relation: Φ = κ_r_ / (κ_r_ + κ_nr_), τ = 1 / (κ_r_ + κ_nr_), where Φ is fluorescence quantum yield and τ is the decay time. κ_r_ is the rate of radiative decay of the singlet state, κ_nr_ is the rate of the non-radiative deactivation of the singlet state.

### Computational Calculations

*Density functional theory (*DFT) and linear response time-dependent density functional theory (TD-DFT) calculations have been performed using the Gaussian 16 package (Frisch et al., 2016). For the calculation of ground and excited state configurations the hybrid exchange-correlation functional M06-2X has been used, unless stated otherwise (Zhao and Truhlar, 2008). This functional is considered suitable for predicting singlet-triplet gaps (Uoyama et al., 2012; Sun et al., 2015). It also offers the possibility to calculate excited state geometries. The 6-31+G(d) atomic basis set has been chosen as it provides a good balance between accuracy and computational cost (Sun et al., 2015). Furthermore, the Tamm-Dancoff approximation (TDA) has been used for more accurate triplet energies (Hirata and Head-Gordon, 1999; Peach et al., 2011).

## Synthesis

### The Tool Box Approach

The aim of this work is to obtain new bipolar and high energy triplet host materials suitable for blue TADF emitters and to consolidate a new molecular strategy that allows to increase the triplet energy of bipolar molecules. For this purpose we introduce a systematic study called tool box approach (Figure 1). Basically, we choose building blocks (donor, acceptor, and linker) that are known to be stable and to have high triplet energies. Practically, triazine was chosen as acceptor group and carbazole and acridine as donor moieties. These groups were combined both with or without linkers obtaining essentially two classes of donor-acceptor molecules (carbazole-triazine and acridine-triazine). For each class we can distinguish between para-phenyl linker, meta-phenyl linker, and without linker. This approach allows us to understand how varying the linker between donor and acceptor affects the final molecule, and so to understand the structure-property relationship. Furthermore, by studying in parallel two classes of molecules, we can demonstrate that via direct linkage between donor and acceptor it is possible to increase the triplet energy above 3 eV and so to create potential bipolar host materials for TADF blue emitters. The reference molecules **pCTRZ** and **pATRZ** were commercially available. The other four molecules (**mCTRZ**, **mATRZ**, **CTRZ**, and **ATRZ**) were synthesized.

**Scheme 1:**
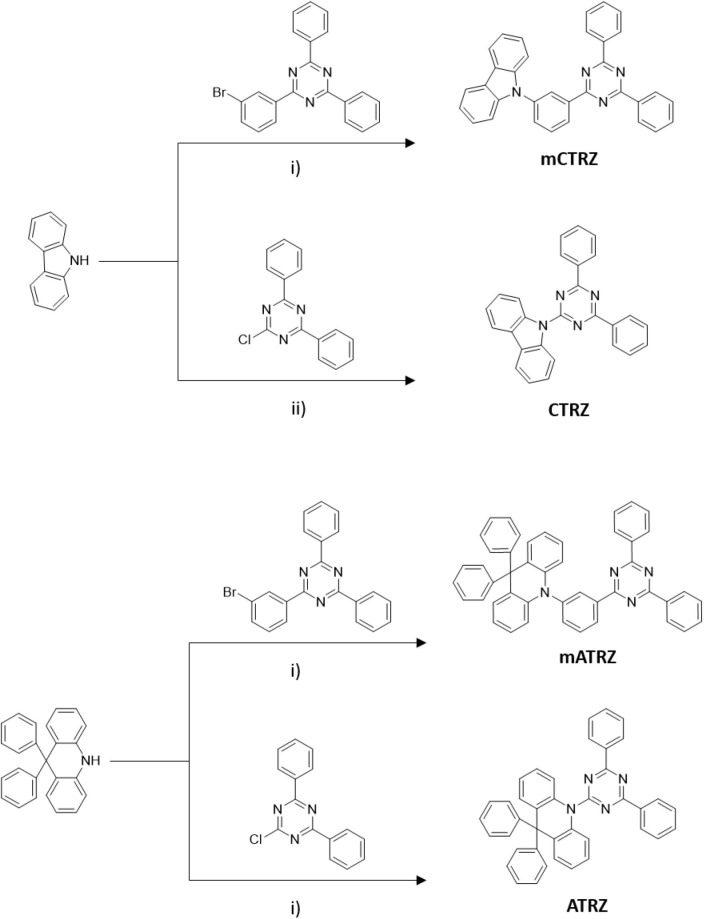
Synthesis routes of the four target compounds. (i) Pd(OAc)_2_, P(tBu)_3_, NaOtBu, toluene. (ii) n-Buli, THF.

The compounds **mCTRZ**, **mATRZ**, and **ATRZ** were synthesized following a general Buchwald-Hartwig protocol, where the NH group comes from carbazole and acridine derivatives, while the halogenated part is contained in the triazine acceptor (or in the meta-linker-acceptor moiety). The compound **CTRZ** can be obtained via simple nucleophilic aromatic substitution of the carbazole with the electron poor triazine ring using nBuLi (Scheme 1). The compounds were obtained with yields from 30 to 57%. **CTRZ** and **mCTRZ** were purified, respectively, via precipitation and column chromatography in a first step. All the compounds were finally purified through vacuum train sublimation.

#### 9-(3-(4,6-diphenyl-1,3,5-triazin-2-yl)phenyl)-9H-carbazole (mCTRZ)

9H-carbazole (259 mg, 1.55 mmol), 2-(3-bromophenyl)-4,6-diphenyl-1,3,5-triazine (500 mg, 1.29 mmol), sodium tert-butoxide (207 mg, 2.15 mmol), palladium acetate (12 mg, 0.05 mmol) and toluene (10 ml) were added to a three necked flask equipped with a stirring bar. The solution was degassed by three freeze-pump-thaw cycles and the flask backfilled with argon. Tri(*tert*-butyl)phosphine in toluene (1 mol/l, 0.1 mmol) was subsequently added to the flask and the mixture refluxed 12 h. After cooling to room temperature, ethyl acetate was added and the solution washed with water. The organic phase was dried over sodium sulfate, filtered and the solvent evaporated under reduced pressure. The pure white product was obtained after purification by column chromatography on silica gel (hexane:dichloromethane = 9:1) and by train sublimation (yield 38%). 1H NMR (CDCl_3_, 500 MHz): δ [ppm] 8.98 (s, 1H), 8.90 (d, J = 7.0 Hz, 2H), 8.76 (d, J = 7.0 Hz, 4H), 8.21 (d, J = 7.5 Hz, 2H), 7.82 (m, 2H), 7.59 (m, 6H), 7.47 (m, 4H), 7.34 (t, J = 7.0 Hz, 2H). 13C NMR (CDCl_3_, 500 MHz): δ [ppm] 171.9, 171.0, 141.0, 138.5, 138.3, 136.0, 132.7, 131.2, 130.3, 129.0, 128.7, 128.1, 127.7, 126.2, 123.5, 120.4, 120.1, 109.8. EI-MS *m/z* [M]^+^: 474.

#### 10-(3-(4,6-diphenyl-1,3,5-triazin-2-yl)phenyl)-9,9-diphenyl-9,10-dihydroacridine (mATRZ)

The synthesis of **mATRZ** was adapted from Lin et al.^[4]^ A reaction set up similar to that of **mCTRZ** is performed by using 9,9-diphenyl-9,10-dihydroacridine (200 mg, 0.6 mmol), 2-(3-bromophenyl)-4,6-diphenyl-1,3,5-triazine (210 mg, 0.54 mmol), sodium tert-butoxide (68 mg, 0.7 mmol), palladium acetate (5 mg, 0.022 mmol), tri(*tert*-butyl)phosphine in toluene (1 mol/l, 0.044 mmol) and toluene (20 ml). After refluxing 12 h, the mixture was filtered through a pad of celite while still hot. Then the filtrate was washed with water and brine, then dried over MgSO_4_ and the solvent evaporated under reduced pressure. The brownish yellow crude product was washed with hexane and dichloromethane and finally purified by train sublimation, obtaining a light yellow solid (yield 30%). 1H NMR (CDCl_3_, 500 MHz): δ [ppm] 8.90 (d, J = 8.0 Hz 1H), 8.73 (d, J = 7.0 Hz, 4H), 8.50 (s, 1H), 7.76 (t, J = 8 Hz, 1H), 7.60 (m, 6H), 7.31 (m, 7H), 7.07 (m, 6H), 6.92 (m, 4H), 6.52 (d, J = 8.0 Hz, 2H). 13C NMR (CDCl_3_, 500 MHz): δ [ppm] 171.8, 170.8, 146.4, 142.1, 141.1, 139.1, 136.0, 135.7, 132.7, 131.9, 130.9, 130.5, 130.0, 129.8, 129.1, 128.8, 128.7, 127.7, 127.0, 126.3, 120.3, 114.1, 56.9. EI-MS *m/z* [M]^+^: 640.

#### 9-(4,6-diphenyl-1,3,5-triazin-2-yl)-9H-carbazole (CTRZ)

**CTRZ** was synthesized according to the procedure reported by An et al. (2011) without using a catalyst. 9H-carbazole (150 mg, 0.9 mmol) and dry tetrahydrofuran (15 ml) were added to a pre-dried three-necked flask under argon atmosphere. The solution was cooled in an ice bath and stirred for 10 min. Then n-butyllithium in hexane (2.5 mol/l, 0.9 mmol) was slowly added and the solution was stirred for 30 min at room temperature. Subsequently, a solution of 2-chloro-4,6-diphenyl-1,3,5-triazine (200 mg, 0.75 mmol) in tetrahydrofuran (5 ml) was added and the mixture was then refluxed for 12 h. The precipitate was filtered and washed with water, acetone and chlorobenzene. The pure white product was obtained after purification by train sublimation (yield 57%). 1H NMR (CDCl_3_, 500 MHz): δ [ppm] 9.17 (d, J = 8.5 Hz, 2H), 8.78 (d, J = 6.5 Hz, 4H), 8.10 (d, J = 8.0 Hz, 2H), 7.64 (m, 8H), 7.45 (t, J = 7.0 Hz, 2H). 13C NMR (CDCl_3_, 500 MHz): δ [ppm] 172.49, 165.26, 139.2, 136.3, 132.8, 129.2, 128.9, 127.0, 126.7, 123.3, 119.7, 117.7. EI-MS *m/z* [M]^+^: 398.

#### 10-(4,6-diphenyl-1,3,5-triazin-2-yl)-9,9-diphenyl-9,10-dihydroacridine (ATRZ)

The synthesis of **ATRZ** was adapted from Lin et al. (2016) 9,9-diphenyl-9,10-dihydroacridine (273 mg, 0.82 mmol), 2-chloro-4,6-diphenyl-1,3,5-triazine (200 mg, 0.75 mmol), sodium tert-butoxide (93 mg, 0.97 mmol), palladium acetate (7 mg, 0.03 mmol) and toluene (20 ml) were added to a three necked flask equipped with a stirring bar. The solution was degassed by three freeze-pump-thaw cycles and the flask backfilled with argon. Tri(*tert*-butyl)phosphine in hexane (10% wt, 0.018 ml, 0.06 mmol) was subsequently added to the flask and the mixture refluxed 12 h. After cooling to room temperature an extraction with water was performed. The organic phase was dried over sodium sulfate, filtered and the solvent evaporated under reduced pressure. The pure light yellow product was obtained after purification by train sublimation (yield 30%). 1H NMR (CDCl_3_, 500 MHz): δ [ppm] 8.36 (d, J = 8.0 Hz 4H), 7.98 (d, J = 8.0 Hz, 2H), 7.51 (7, J = 7.0 Hz 2H), 7.45 (t, J = 7.5 Hz, 6H), 7.24 (t, J = 7.5 Hz 2H), 7.03 (m, 8H), 6.88 (m, 4H). 13C NMR (CDCl_3_, 500 MHz): δ [ppm] 168.5, 162.3, 141.4, 140.8, 137,4, 134.4, 129.6, 129.6, 126.5, 126.1, 126.0, 125.3, 125.2, 124.5, 123.6, 122.8, 56.6. EI-MS *m/z* [M]^+^: 564.

Except for **mATRZ** and **ATRZ**, all compounds have already been investigated in some part, either experimentally, or by calculations, or both (An et al., 2011, 2015; Lin et al., 2016; Duan et al., 2018; Arjona-Esteban et al., 2019; Fan et al., 2019; Liu et al., 2019; Sharma et al., 2019). These previous investigations do not form a complete and consistent data set, and sometimes are even contradictory. We therefore decided to perform a systematic spectroscopic and theoretical study that is conducted for all compounds under identical conditions.

## TD-DFT-Analysis

### Choice of Functional

The calculation of excitation energies for donor-acceptor type molecules with charge-transfer characteristic can be a challenge for density functional theory calculations (Sun et al., 2015). In order to obtain results with good accuracy, we therefore first performed a study on the suitable choice of the functional. For this, we employed six popular exchange-correlation functionals on the molecule **CTRZ** which has been well-characterized experimentally in previous works (An et al., 2011; Duan et al., 2018) as well as in this work. We used two range-separated hybrid functionals, CAM-B3LYP (Yanai et al., 2004) and ωB97XD (Chai and Head-Gordon, 2008), three hybrid meta-GGA functionals, M06-2X (56% exact exchange), M06-HF (100 % exact exchange) (Zhao and Truhlar, 2008) and PW6B95D3(28% exact exchange) (Zhao and Truhlar, 2005) and a meta-GGA functional, PBE0(25% exact exchange) (Adamo and Barone, 1999). Table 1 compares the calculated S_1_ and T_1_ emission energies, in the relaxed excited state geometry, obtained for **CTRZ** with these 6 functionals with the experimentally measured value (see next section). The best match between experiment and calculation for the S_1_ energy is found for the functional CAM-B3LYP, closely followed by M06-2X. However, for the T_1_ energy, which is most important to us, M06-2X is closest to the experimental value. We therefore decided to calculate the S_1_ and T_1_ excitation energies for all 6 molecules with M06-2X, and, for reference, also with the widely employed functional CAM-B3LYP. Table 2 compares the resulting energies with the experimental values all taken from the subsequent section “spectroscopic analysis.” While all calculated values are in good agreement with the experiment, the excellent match obtained for the triplet energies using M06-2X is noteworthy. Even though this functional is considered suitable for predicting singlet-triplet gaps (Uoyama et al., 2012; Sun et al., 2015), the agreement between calculated values and experimental ones is lessened for the singlet-triplet gap. Results for the functional CAM-B3LYP can be found in the supporting information (Table S1).

**Table 1:** Emission energies as well as their difference (in eV) in optimized S_1_ and T_1_ geometries for the molecule **CTRZ** calculated with different exchange-correlation DFT functionals and a 6-31+G(d) basis set.

	**S_**1**_**	**T_**1**_**	**Δ(S_**1**_ – T_**1**_)**
Experiment	3.21	2.97	0.24
M06-2X	3.18	3.00	0.18
CAM-B3LYP	3.26	2.77	0.49
M06-HF	3.57	3.28	0.29
PBE0	2.29	2.31	−0.02
PW6B95D3	2.36	2.38	−0.02
ωB97XD	3.51	2.85	0.66

*The experimental value is taken from the spectroscopic analysis in this paper*.

**Table 2:** Emission energies (in eV) in optimized S_1_ and T_1_ geometries calculated at the M06-2X/6-31+G(d) level.

	**S_1_^a^ exp. (eV)**	**T_1_^b^ exp. (eV)**	**ΔE_**ST**_, exp. (eV)**	**S_**1**_ (M06-2X)**	**T_**1**_ (M06-2X)**	**ΔE_**ST**_ (eV)**
**pCTRZ**	3.22	2.76	0.46	3.48	2.69	0.79
**mCTRZ**	3.21	2.82	0.39	3.44	2.82	0.62
**CTRZ**	3.21	2.97	0.24	3.18	3.00	0.18
**pATRZ**	2.92	2.67	0.25	3.06	2.60	0.46
**mATRZ**	2.94	2.72	0.22	3.02	2.92	0.10
**ATRZ**	3.25	3.07	0.18	3.33	3.03	0.30

*For comparison, the experimental values are also given for molecules in toluene at 77 K, as derived below*.

a *The energy of the singlet state was determined by fitting the spectral lineshape of the CT absorption and fluorescence, **Figure 4***.

b *The energy of the triplet state was taken from the 0-0 the position of the phosphorescence at 77 K*.

We next considered how the natural transition orbitals (NTO) obtained with M06-2X for our reference molecule **CTRZ** compare to those obtained by other functionals. The NTOs obtained for the ground state S_0_ geometry of **CTRZ** for M06-2X are shown in the supporting information (Figure S2). The orbitals are in excellent agreement with the ones from Duan et al. (2018) who used the range-separated functional LC-ωPBE. Finally, we calculated and compared the NTOs in the excited state S_1_ geometry for all 6 functionals (Figure S3). Five out of six functionals show a strong charge transfer character for the S_1_ state and a localized excitation for the T_1_ state, among them M06-2X, suggesting that this is likely to be the prevailing scenario (as will be confirmed on the basis of the spectroscopic data further below).

### Ground State Geometries and Excited State NTOs

**Figure 2:**
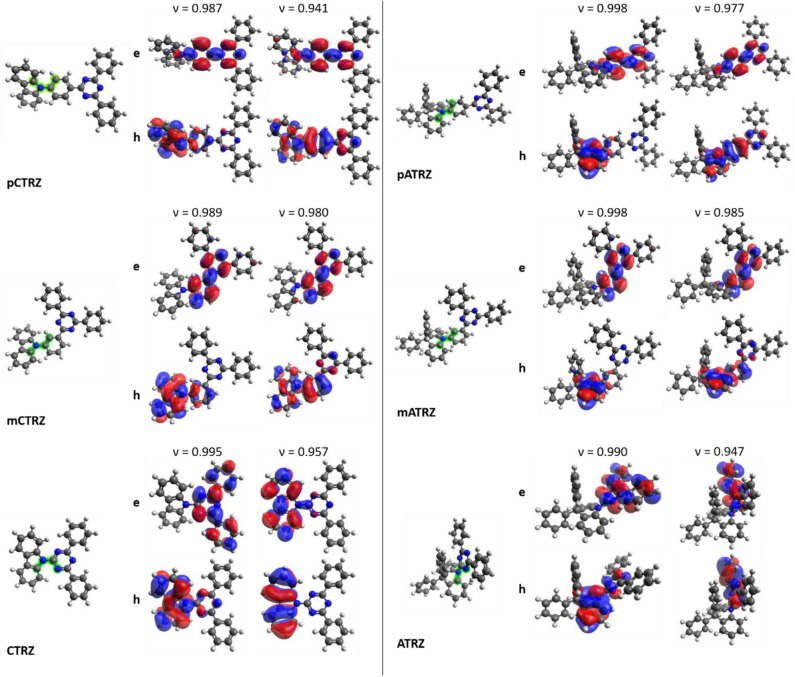
Electronic and structural properties of the investigated molecules derived from DFT/TD-DFT calculations at the M06-2X/6-31+G(d) level. Optimized ground state S_0_ structures of the molecules with the investigated dihedral from Table 1 highlighted in green are shown next to the dominant NTOs of the molecules in relaxed singlet S_1_ and triplet T_1_ state structure. Top images represent the electron density e and bottom images the hole density h. The corresponding weight *v* of the NTO is also given.

Figure 2 shows the molecules in the relaxed S_0_ ground state geometry, alongside the dominant NTOs for the S_1_ and T_1_ in the relaxed excited state geometry. It is well-known that the electronic coupling between the donor and acceptor depends strongly on the dihedral angle between them. This angle is indicated in Figure 2 in green color and detailed in Table 3. We find that **pCTRZ** and **mCTRZ** are strongly twisted with practically the same value of about 52°, while **CTRZ** is more planar. Similarly, for the acridine based molecules **pATRZ** and **mATRZ** we observe an almost orthogonal configuration between donor and acceptor moiety while **ATRZ** shows essentially no twist between donor and acceptor though it has a bended shape of acridine core. When going from the ground to the excited states, we find an overall more planar geometry for the T_1_ state, while the S_1_ state becomes even slightly more twisted for the carbazole-derivatives. For the **pATRZ** and **mATRZ**, the S_1_ geometry remains more or less unaltered, and it planarizes for the **ATRZ**.

**Table 3:** Dihedral angles (in degree) between the donor and acceptor unit in the ground and excited state geometry calculated at the M06-2X/6-31+G(d) level.

	**S_**0**_ (^**°**^)**	**S_**1**_ (^**°**^)**	**T_**1**_ (^**°**^)**	***f***	**S_**1**_ (eV)**
**pCTRZ**	52	66	43	0.5872	3.91
**mCTRZ**	53	56	40	0.0170	3.93
**CTRZ**	19	40	13	0.0071	4.21
**pATRZ**	97	93	55	0.0005	3.66
**mATRZ**	100	86	62	0.0002	3.73
**ATRZ**	14	2	10	0.0129	4.47

*In case of asymmetry of the molecule only the smaller dihedral is shown. The oscillator strength f for the S_*0*_ → S_*1*_ transition is also given, along with the energy for the vertical transition from the relaxed ground state geometry*.

The NTOs of the molecules in the optimized lowest singlet S_1_ and triplet T_1_ excited state geometry deserve some consideration. For all molecules, the calculations predict that the S_1_ → S_0_ transition is accompanied by an electron transfer from the acceptor part of molecule to the donor unit, though there is also some overlap of differing degree on the phenyl bridge or triazine ring. This implies a mixed nature of the transition, with a dominant charge transfer (CT) character, in agreement with conclusions published earlier for some of the compounds (An et al., 2011; Lin et al., 2016; Duan et al., 2018; Fan et al., 2019; Liu et al., 2019). The oscillator strengths for this transition is given in Table 3. From the para to the meta-connection, the oscillator strength reduces significantly, while there is no further significant reduction when going from **mCTRZ** to **CTRZ** and even a small increase from **mATRZ** to **ATRZ**.

Regarding the triplet T_1_ state, we find a pronounced difference between the molecules with a p-phenyl or m-phenyl bridge and the molecules without a linker between donor and acceptor. With the phenyl bridge, and in particular for the *p*-phenyl, the electron and hole orbitals of T_1_ demonstrate a somewhat stronger overlap than for the S_1_ state, even though some charge transfer still takes place. In previous works, the character of the T_1_ state was therefore interpreted either as a CT state with strong overlap of electron and hole orbitals at the triazine, phenyl bridge, and the amino part of the carbazole unit, (Fan et al., 2019) or as a localized excitation (LE) spread over the triazine, phenyl bridge, and carbazole unit (Sun et al., 2015). It was also described as a superposition of the CT state with an LE state in the acceptor (Duan et al., 2018). Our calculations confirm the general mixed CT-LE character of the *p*-phenyl and *m*-phenyl bridge donor-acceptor compounds, with a stronger LE contribution for T1 than for S1.

In contrast to this, we find a strongly LE character for the T_1_ state of the molecules without bridge, i.e., **ATRZ** and **CTRZ**, with the excitation fully on the donor for **CTRZ** and fully on the acceptor for **ATRZ**. For **CTRZ**, this differs from the prediction of Duan et al. who predicts a strong contribution from orbitals delocalized over the entire molecule (Duan et al., 2018). We attribute this difference to the fact that Duan et al. based their calculations on the ground state geometry, while we were considering the relaxed T_1_ excited state geometry. The calculations predict the triplet from this confined LE state to be at higher energy than the triplet from the molecules with the phenyl ring bridge.

## Spectroscopic Analysis

### Absorption and Fluorescence in Toluene Solution at Room Temperature

**Figure 3:**
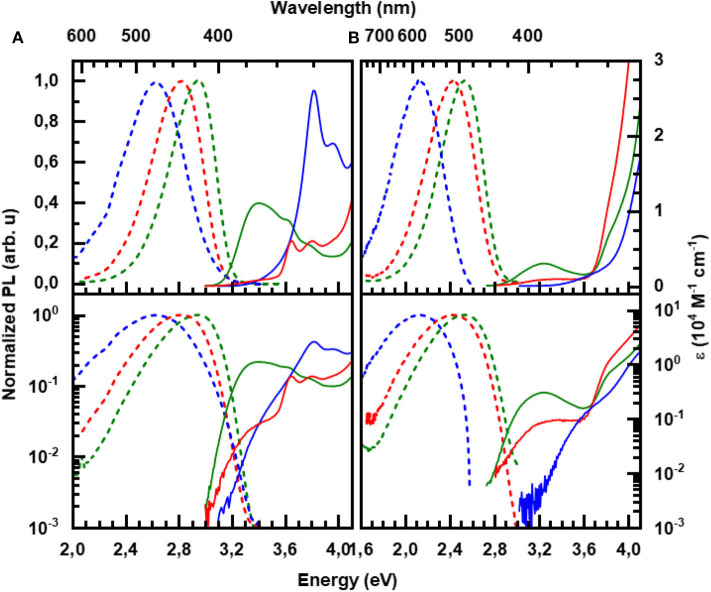
Absorption (solid lines) and steady state emission (dashed lines) at room temperature for **(A)** the carbazole-based and **(B)** the acridine-based series in toluene solution: **pCTRZ** and **pATRZ** (green), **mCTRZ** and **mATRZ** (red), and **CTRZ** and **ATRZ** (blue). The concentration of the solutions is 0.05 mg/mL, excitation was at 350 nm (3.54 eV). The bottom panel displays the same data on a logarithmic scale.

The absorption spectra of our 6 molecules under investigation in toluene at low concentration are shown in Figure 3. The spectra are consistent with those published previously (An et al., 2015; Lin et al., 2016; Fan et al., 2019; Liu et al., 2019). For reference, the spectra of the constituent units, carbazole, diphenyltriazine, and diphenylacridine, are displayed in the supporting information (Figure S1).

The absorption of **pCTRZ** is characterized by presence of strong broad band in the range 3.1–4.1 eV with maximum at 3.41 eV, followed by structured features at 3.63 and 3.80 eV. Comparison with the absorption spectrum of carbazole (Figure S1) readily identifies the features at 3.63 and 3.80 eV as resulting from a π – π ^*^ transition (and its vibrational replica) localized on the carbazole moiety. When the para-connection is replaced by a meta-linkage, as in **mCTRZ**, the features remain at the same energy yet the extinction of the broad band at 3.41 eV reduces by about one order of magnitude, as is evident from the display on the logarithmic scale (bottom panel of Figure 3). This band at 3.41 eV is commonly interpreted as corresponding to CT transition, (Cui et al., 2016b; Fan et al., 2019) though the high absorption for **pCTRZ**, consistent with the high calculated oscillator strength of about 0.6 (see Table 3, and of 0.3 by Fan et al., 2019) suggests a strong influence of the π – π ^*^ transitions from the carbazole. This is further supported by the drastic reduction in the extinction coefficient and calculated oscillator strength when reducing the conjugation through the meta-linkage in **mCTRZ**. It is remarkable that the influence of the para- and meta-connection on the CT-dominated absorption band is only reflected in the band's intensity, yet not in the energetic positions of either the CT-dominated band or the π – π^*^ transitions from the carbazole. In **CTRZ**, where the phenyl linker is entirely omitted this is different. The direct connection of carbazole to diphenyltriazine shifts the carbazole π – π^*^ band to higher energy by about 0.15 eV, and increases its oscillator strength. Compared to carbazole (Figure S1), the low energy edge has a pronounced tail. On the logarithmic scale, a superimposed broad transition centered at about 3.5 eV with similar extinction than the CT band in mCTRZ becomes evident, suggesting that the 3.5 eV feature is the CT band in CTRZ. Thus, it is evident that the extinction coefficient of the CT band decreases from **pCTRZ** to **mCTRZ** and **CTRZ**, roughly consistent with the calculated oscillator strengths of 0.587, 0.017, and 0.007, and suggestive of a reduced wavefunction overlap between donor and acceptor in **mCTRZ** and **CTRZ** compared to **pCTRZ**.

The acridine-based series (Figure 3B) shows some similarities in their general spectroscopic properties to the carbazole-based series. Like **pCTRZ** and **mCTRZ**, **pATRZ** and **mATRZ** show a broad, weak transition centered at 3.24 eV for both compounds, that reduces in intensity when the para-connection is replaced by a meta-linkage. Based on the broad structureless shape and its low extinction coefficient, we attribute this band to a CT-transition, consistent with the NTOs obtained in the S_0_ geometry (Figure S4). Analogous to **CTRZ**, for **ATRZ**, this CT band is blue shifted by about 0.15 eV and similar in intensity to the CT band in **mATRZ**. The transitions between 3.6 and 4.0 eV can be attributed to the n – π^*^ transitions of the acridine chromophore that dominate over the n– π^*^ transition of triazine by comparison with the absorption of the constituent units (Figure S1). Above 4 eV, transitions of the solvent toluene take over. A pronounced difference to the carbazole-based series is the lower extinction of the CT bands. This can be credited to the different geometry of the phenyl-linked molecules in the ground state. As detailed in Table 3, for **pCTRZ** and **mCTRZ**, the angle between donor and acceptor is 52 and 53°, while for **pATRZ** and **mATRZ**, it is 97 and 100°, respectively. As a result, the donor and acceptor in these acridine compounds are more decoupled, consistent with the S_0_ NTOs (Figure S4). Conversely, the more planar structure in **ATRZ** thus leads to still sizable oscillator strength and some wavefunction overlap despite the general donor-acceptor nature of this compound (c.f. Figure S4).

Figure 3 presents also the room temperature emission spectra of the compounds in the toluene solution. As detailed in Table 4, the emission decays within a few ns for the carbazole-series and within a few tens of ns for the acridine-series, thus identifying it as fluorescence. For both series, the PL QY reduces along the series. When calculating the decay rates the higher radiative rate for the para-connected compounds becomes evident as cause for the relatively high PLQYs, which are more typical for π – π^*^ emission than CT emission. This is in agreement with the trends in wavefunction overlap for the relaxed S_1_ state (c.f. Figure 2). The meta-linked compounds and compounds without phenyl connection have similarly low radiative decay rates. However, when the phenyl ring is omitted, the non-radiative decay rate increases. We suggest that in **CTRZ** and **ATRZ** the torsional modes enhance non-radiative decay routes, notably intersystem crossing (ISC) (Beljonne et al., 2001).

**Table 4:** PL Quantum yield and lifetime for the compounds in x mg/ml toluene at 300 K.

**Compound**	**QY**	**τ (ns)**	**k_**r**_, (10^**8**^ s^**−1**^)**	***k*_**nr**_ (10^**8**^ s^**−1**^)**
**pCTRZ**	0.85	3.8^a^	2.40	0.40
**mCTRZ**	0.15	18.6^a^	0.08	0.46
**CTRZ**	0.04	6.2^a^	0.06	1.56
**pATRZ**	0.19	13 (99%), 52 (1%)^b^	0.15	0.64
**mATRZ**	0.05	25 (97%), 90 (3%)^b^	0.02	0.38
**ATRZ**	0.03	12 (80%), 35 (20%)^b^	0.02	0.81

*The radiative and non-radiative decay rates, k_r_, and k_nr_, are derived as detailed in the experimental section. To avoid any contribution from delayed fluorescence or phosphorescence, the solution was saturated with air*.

a *Taken from an exponential fit to the decay curve (Figure S8)*.

b *Taken from a biexponential fit to the decay curve (Figure S8). The values in bracket give the weight of the two exponential decay*.

**Figure 4:**
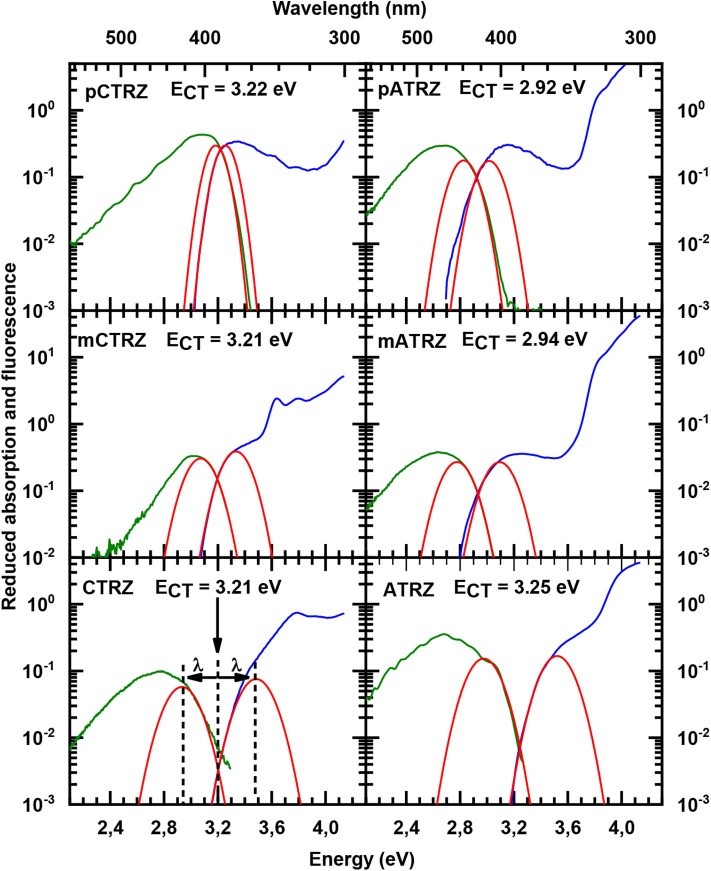
Reduced absorption (blue lines) and reduced fluorescence (green lines) at 77 K in toluene for **pCTRZ**, **mCTRZ**, **CTRZ**, **pATRZ**, **mATRZ**, and **ATRZ**. Red lines correspond to Gaussian fits as described in the text.

For both series, we find that the peak of the CT absorptions coincides for the compounds with a para or meta-connected phenyl ring, and it is 0.15 eV more blue if the phenyl ring is omitted. In contrast, the fluorescence peaks are distinct for within a series, and the peak energies decrease from para-linked to meta-linked followed by no link. Clearly, for such structureless spectra the determination of excited state energy is a challenge. Using the onset of the fluorescence or absorption spectra is a frequently used approach, but it carries a large uncertainty. A more precise value can be obtained by employing an approach that is common to determine the CT state energy in compounds used for organic solar cell applications, first introduced to the field by Vandewaal and co-workers (Gould et al., 1993; Vandewal, 2016; Kahle et al., 2018; Vandewal et al., 2010, 2017). For this, absorption and emission are plotted as reduced absorption and reduced emission spectra, i.e., the absorption (already displayed in energy intervals) is multiplied by the photon energy, and the emission (also already displayed in energy intervals) is divided by photon energy. The high energy edge of the emission and the low energy edge of the absorption are then fitted with the same gaussian lineshape. The intersection of the two curves indicates the position of the CT state 0-0 transition energy. The energy difference between the intersection and the maxima of the gaussian lineshape gives the reorganization energy associated with the CT state. The presentation of the spectra in this form is shown in Figure 4. The values obtained this way are presented in Table 2. One can see that such an analysis gives essentially the same CT state energy for all three carbazole-based molecules. They only differ in the value of the associated reorganization energy that increases along the series. Regarding the acridine-based series, the same CT state energy is obtained for **pATRZ** and **mATRZ**. while the **ATRZ** compound has its CT band blue-shifted to them and also shows a significantly larger reorganization energy.

**Figure 5:**
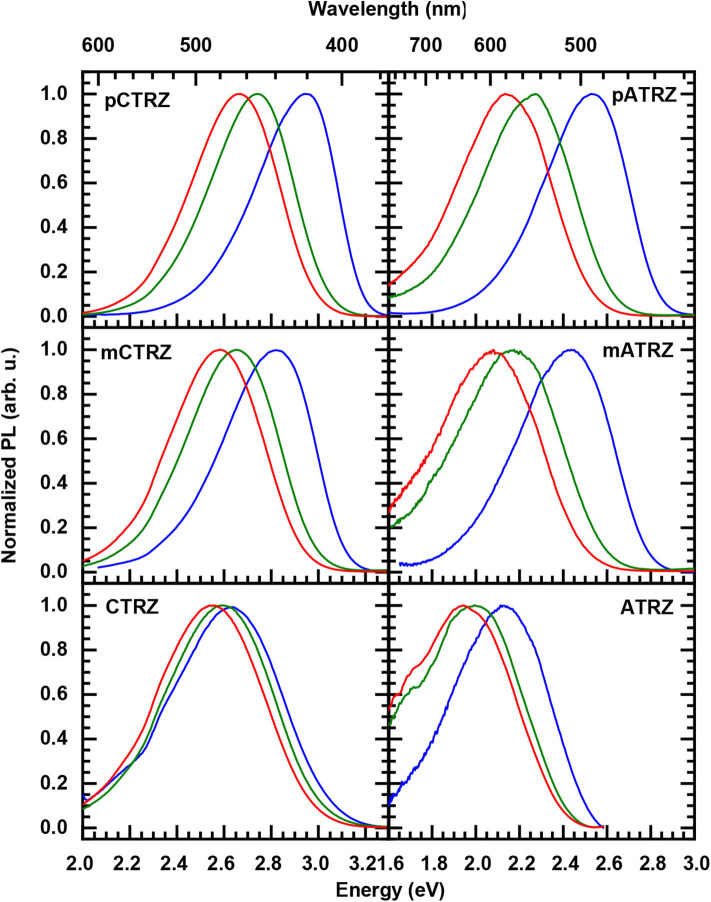
Solvent-dependent 300 K steady state emission spectra of **pCTRZ** and **pATRZ**, **mCTRZ** and **mATRZ**, **CTRZ** and **ATRZ** in toluene (blue lines), THF (green lines), and DCM (red lines). Concentration of the solutions is 0.05 mg/mL. Excitation is at 350 (3.54 eV) nm.

This larger reorganization energy for **CTRZ** and **ATRZ** can be due to intra-molecular reorganization, e.g., associated with the change in torsion angle between the S_0_ and S_1_ geometry (Table 3) that prevails for CTRZ and ATRZ, and that does not occur to a similar extent in the compounds linked by a phenyl ring. The reorganization energy usually includes both, contributions from changes in the molecular geometry as well as contributions from solvent reorganization. At 77 K, the solvent is frozen, implying that solvent reorganization is very limited. At room temperature it will, however, also include strong contributions from the solvent reorganization that reflect different strength in the CT character of the excited state. The larger reorganization energy for **ATRZ** is therefore likely to involve intra-molecular rearrangements. This can be assessed by considering the fluorescence taken in solvents of different polarity, such as toluene, tetrahydrofurane (THF), and dichloromethane (DCM), which have relative polarities of 0.099, 0.207, and 0.309, respectively (Reichardt, 2002) (Figure 5). We observe a weaker solvent dependence of the fluorescence peaks in **CTRZ** and **ATRZ** compared to the phenyl-bridged compounds. This seems counter-intuitive, as it would suggest a weaker CT-character. At closer inspection, one notices that in the most polar solvent, DCM, where the CT-state can be expected to be most stabilized, the fluorescence of all compounds within a series roughly coincide in their peak position. For less polar solvents, the emission from the meta-linked and even more from the para-linked compounds is blueshifted, implying a reduced stabilization from a lesser CT-character of the excited state. The fact that **CTRZ** and **ATRZ** have a large reorganization energy even in less polar solvent therefore suggests that the dominant contribution to its reorganization is due to the change in molecular geometry such as the increased dihedral angle predicted by the TD-DFT calculation (Table 3). We recall that the TD-DFT calculations are carried out for the gas phase, and that more sophisticated calculations (beyond the scope of this paper) including the effect of solvent polarizability would be required to address this issue in a quantitative manner.

### Fluorescence and Phosphorescence at 77 K

**Figure 6:**
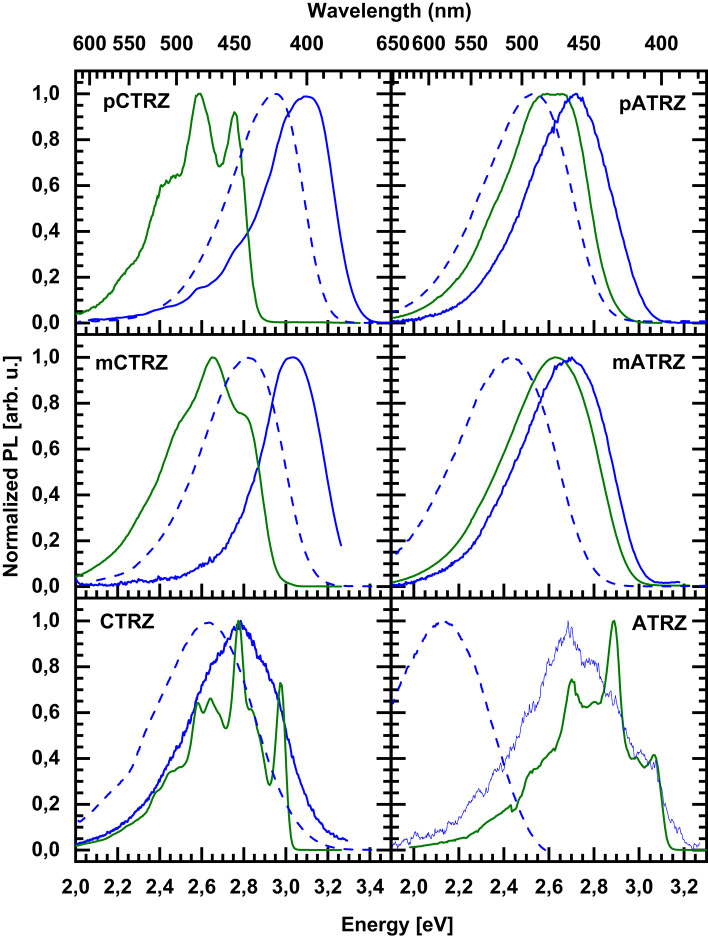
Emission spectra taken at 77 K in toluene of **pCTRZ**, **mCTRZ**, **CTRZ**, **pATRZ**, **mATRZ**, and **ATRZ** at a concentration of 0.05 mg/mL. Green lines correspond to phosphorescence, blue ones to fluorescence. Dashed lines show fluorescence at room temperature.

We turn to measurements taken at 77 K, displayed in Figure 6. Upon cooling, the fluorescence spectra shift to the blue spectral range. The shifts are 0.15, 0.20, and 0.15 eV along the carbazole-based series, and 0.20, 0.30, and 0.60 eV for the acridine-based series. We attribute the hypsochromic shift to the freezing out of molecular motion. The solvent shell molecules can no longer reorient after the transition of the molecule to the excited state, thus precluding the stabilization of the CT state. Based on the (gas phase) TD-DFT calculations (Table 3) and the polarity dependence observed in Figure 5 it seems that for **CTRZ** and **ATRZ**, there is also a contribution from the impediment of structural changes of the emitter molecules after excitation, such as changes in the dihedral angle, at 77 K.

Regarding the phosphorescence, we are interested to experimentally identify not only the S_1_-T_1_ gap, but also the part of the molecule from which the transition originates. We associate the T_1_ energy with the position of the 0-0 phosphorescence peak. For the carbazole-based series and for **ATRZ**, these can be clearly identified as 2.76, 2.82, 2.97, and 3.07 eV. For **pATRZ**, the 0-0 peak is just resolvable at 2.67 eV. The spectral resolution is poorer for **mATRZ**. Comparing the spectra of **pATRZ** and **mATRZ** (see Figure S5), we find that **mATRZ** is overall shifted to the blue by 0.05 eV, so we estimate the 0-0 position to be at about 2.72 eV. All triplet energies are summarized in Table 2.

**Figure 7:**
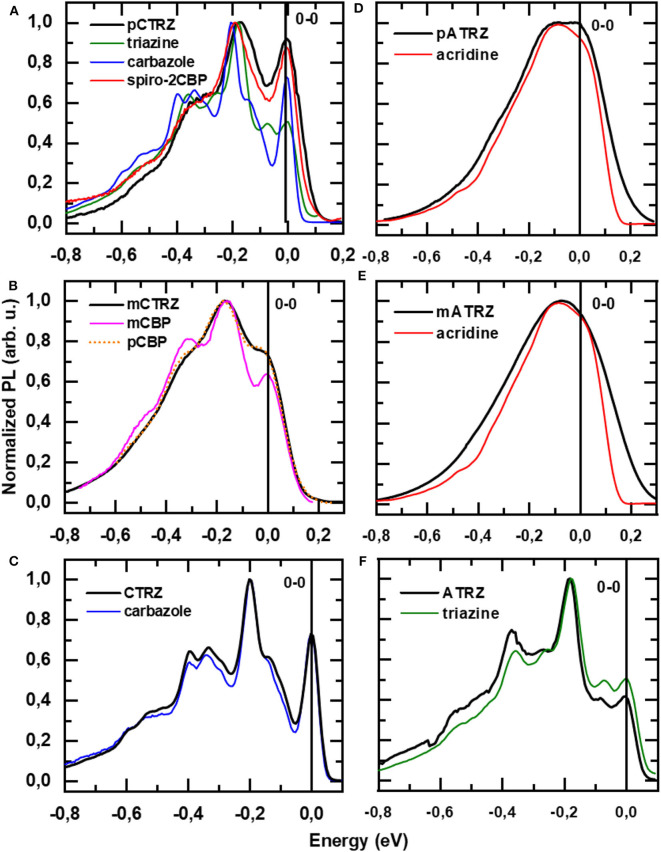
**(A)** Phosphorescence spectra **pCTRZ (A)**, **mCTRZ**, **(B) CTRZ (C)**, **pATRZ (D)**, **mATRZ (E)**, and **ATRZ (F)** and corresponding modeling compounds in toluene at 77 K. The energy scale for every compound is adjusted to coincide to the first energy maximum. All compounds except triazine were excited at 350 nm, and emission was detected after a 30 ms delay with an integration of 15 ms. Triazine solution was excited at 300 nm.

In order to identify the origin of the T_1_ state, we compare the vibrational structure of the phosphorescence spectra of our two series with that from their molecular constituents (Figure 7). To ease comparison, all spectra are shifted in energy so that the 0-0 positions coincide at 0 eV. The original spectra are given in the supporting information (Figure S6). It is immediately evident that the spectra of **CTRZ** and of **ATRZ** coincide with the spectral form obtained from only a carbazole or triazine. The observation that the vibrational sidebands coincide implies that the transition is localized on the carbazole moiety in the case of **CTRZ** and on the triazine moiety in the case of **ATRZ**. In fact, even the energies of the transitions agree well (Figure S6). This spectroscopic result is in excellent agreement with the TD-DFT calculations and, for **CTRZ**, at variance with earlier suggestions that involve transitions delocalized over the entire **CTRZ** molecule (Duan et al., 2018). A different picture results for the compounds that are bridged by a phenyl ring. The vibrational structure in the phosphorescence of **pCTRZ** and **mCTRZ** resembles neither that observed in carbazole nor that of triazine. Rather, it closely resembles the structure obtained for carbazole-biphenyl derivatives, where the triplet state was found to be localized on the central two phenyl rings (Brinen et al., 1966; Bagnich et al., 2015a,b). Thus, we conclude that the triplet state is dominated by the transition localized on the phenyl bridge and the triazine core, again in very good agreement with the theoretical predictions. For **pATRZ** and **mATRZ**, the spectra are broad and differ drastically from the phosphorescence spectra of **pCTRZ** and **mCTRZ**. Rather, they are close in shape to spectrum of the donor, 9,9-diphenyl-9,10-di-hydroacridine. Spectra with practically the same shape were observed for other donor-acceptor materials containing acridine (Liu et al., 2015, 2016a,b). It allows to infer that triplet state localization includes also donor molecule, as also implied by the DFT calculations. Overall, we can summarize that for the compounds without bridge, the triplet state is localized strictly on the donor or acceptor moiety, while for the compounds with the phenyl bridge, the orbitals involved in phosphorescence spread from the center of the molecules into adjacent moieties.

We note that within a series the relative intensity of the phosphorescence differs. In the case of **pCTRZ** and **pATRZ**, phosphorescence is so weak that it does not contribute in the low temperature steady-state luminescence (Figure S7). For meta**-**derivatives the steady-state luminescence consists mostly from the phosphorescence, though some fluorescence is still observed as blue shoulder. Finally, only phosphorescence is observed in the steady-state luminescence of **CTRZ** and **ATRZ**. The data presented in Table 4 allow to understand the different contribution of the phosphorescence. In the case of **pCTRZ** the main channel of the singlet state deactivation is radiative decay with a rate that is 6 times larger than rate of non-radiative decay, that includes internal conversion and ISC. In this case ISC cannot compete with radiative decay of the singlet state that responsible for intensity of fluorescence. Change of carbazole position in the molecule from para to meta or removing of the phenyl bridge lead to a strong (about 30–40 times) decrease of value for radiative decay rate, so that the non-radiative processes, in particular the ISC, dominate. This is particularly strong for **CTRZ**, where the non-radiative rate increases compared to **mCTRZ**. The same trend takes place for acridine based compounds.

## Discussion and Summary

The aim of our “toolbox approach” was to understand how to maximize the triplet energy in donor-acceptor type bipolar compounds through the choice of connection. We find that the use of a para-phenyl bridge or a meta-phenyl bridge both moves the lowest energy triplet state onto the center of the molecule so that its orbitals expand over more than two rings. This lowers the triplet state energy. This finding is consistent with earlier observations by us made on carbazole-biphenyl type molecules that do not contain any donor-acceptor type interactions, as well as with studies by Brunner and coworkers on carbazole derivatives, who emphasized the strong dependence of the triplet state energy on the number of aromatic rings over which the triplet wavefunction can spread (Brunner et al., 2004; Bagnich et al., 2015a,b). Thus, the lower triplet energy relates predominantly to the structure of the connection that allows for the triplet to spread over more than two rings. Similarly, when the phenyl bridge is omitted, as in **CTRZ** and **ATRZ**, the triplet is strongly localized on the smaller donor or acceptor unit, and thus has a high triplet energy, characteristic for small molecular units. The highest triplet energy in our series, 3.07 eV for **ATRZ**, is consequently also found for the molecule where the triplet is confined onto the phenyl triazine moiety, as confirmed by the vibrational structure and the TD-DFT calculations (Figure 2), see also Figure S10 for a different projection of **ATRZ**). We note that this is one of the highest triplet energies reported so far for potential OLED bipolar host materials. Our results further suggest that obtaining higher triplet energies would imply localizing the triplet on an even smaller unit.

There are two further points emerging from our study that are worth commenting on. First, we recall the stronger dependence of the fluorescence maximum on solvent polarity that we observed for the phenyl-bridged compounds, even though they have ππ^*^ admixtures in their excited state wavefunction, compared to the non-bridged compounds **CTRZ** and **ATRZ** that have a dominant CT character. Evidently, this implies a caveat that the lack of a strong shift with solvent polarity is not an unambiguous proof for a weak CT character, as it can be masked by effects due to conformational changes.

Second, our study illustrates the difficulty in defining a measure for the excited state energies. When the fluorescence and phosphorescence spectra are well-structured, so that the 0-0 emission peak can be clearly identified, quoting the energy of that peak is a very good approach, if not even the best practice. Difficulties arise if one of the spectra is poorly structured. We determined the S1 energy by spectral fitting as is common for CT states in organic solar cells (Gould et al., 1993; Vandewal et al., 2010, 2017; Vandewal, 2016; Kahle et al., 2018). One alternative is to consider the onset of fluorescence and phosphorescence, identified by extrapolating the slope of the high energy tail. Tacitly, this adds the sum of the linewidth to the transition energy and it bears the danger of misjudging singlet-triplet gaps if the linewidth differs between fluorescence and phosphorescence. If the high-energy edges of the fluorescence and phosphorescence have comparable slopes, implying comparable linewidth, taking the difference between the high-energy edges, e.g., at half maximum, can be an approximation to obtain the energy splitting between the two transitions without too much error (see also, Table S2).

In summary, using a tool box approach we synthesized and investigated six bipolar host materials that vary in the way how donor and acceptor are connected. Our detailed photophysical characterization supported by quantum chemical calculations show how this connection controls the excited states and their energies. Only direct connection of the donor group to the triazine core provides a high energy of the triplet state (2.97 eV for **CTRZ** and 3.07 eV for **ATRZ** in toluene), which is of prime importance for the use of the materials as a host for blue TADF emitters.

## Data Availability Statement

The datasets generated for this study are available on request to the corresponding author.

## Author Contributions

FR conducted the synthesis and characterization of the materials. ED and SB carried out and interpreted the photophysical measurements. TM, SA, and JK were responsible for the DFT calculations. AK and PS supervised the experiments and corrected the manuscript. All authors contributed to the article and approved the submitted version.

## Conflict of Interest

The authors declare that the research was conducted in the absence of any commercial or financial relationships that could be construed as a potential conflict of interest.
