# Supplementary material for: High Triplet Energy Host Materials for Blue TADF OLEDs—A Tool Box Approach
Source: Front Chem. 2020 Jul 29;8:657. doi: 10.3389/fchem.2020.00657 (PMC7403631; doi:10.3389/fchem.2020.00657)
Supplement: Supplementary file 1 [file Table_1.docx]

**Supplementary information for:**

**High triplet energy host materials for blue TADF OLEDs – A tool box approach**

Francesco Rodella,^a#^, Sergey Bagnich,^b#^, Eimantas Duda,^b^ Tobias Meier, ^b^ Julian Kahle, ^b^ Stavros Athanasopoulos,^c^ Anna Köhler^b,d,*^ and Peter Strohriegl^a,d,*^

^a^ Macromolecular Chemistry I, University of Bayreuth, Bayreuth 95440, Germany

^b^ Soft Matter Optoelectronics, University of Bayreuth, Bayreuth 95440, Germany

^c^ Departamento de Física, Universidad Carlos III de Madrid, Avenida Universidad 30, Leganés 28911, Madrid, Spain

^d^ Bayreuth Institute of Macromolecular Research (BIMF), University of Bayreuth, Bayreuth 95440, Germany

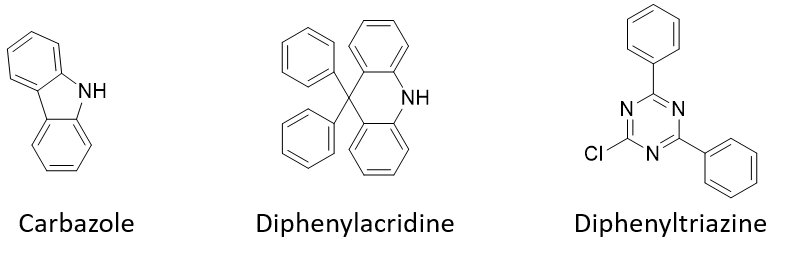


Figure S1. Absorption of the diphenylacridine, carbazole and diphenyltriazine units and their molecular structure.

Figure S2. NTOs and weights (ν) of CTRZ for S_1_ and T_1_ in the ground state geometry calculated at the M06-2X/6-31+G(d) level. The lower image shows the hole density (h), the upper one the electron density (e).

Figure S3. NTOs and weights (ν) of S_1_ and T_1_ excited state structures calculated with different functionals as indicated for the molecule CTRZ. NTOs and geometric optimization have been calculated with the same functional. The basis set is always 6-31+G(d). Top images represent electron density (e) and bottom images the hole density (h). For the S_1_ state a clear charge transfer character is observed for all functionals except for M06-HF with the hole density being on the carbazole unit and the electron density on the triazine unit. The electron and hole wave function of the T_1_ state are almost completely localized on the carbazole unit with the exception of PBE0 which shows a strong charge transfer character. In each state, S_1_ and T_1_, the NTOs give the same result for five out of six orbitals suggesting that this is the actual scenario.

Figure S4. NTOs of the molecules for the S_0_ state calculated from DFT at the M06-2X/6-31+G(d) level in the relaxed ground state geometry. Top images represent the electron density (e) and bottom images the hole density (h), alongside with the corresponding weight ν.

Figure S5. Phosphorescence of **pATRZ** and **mATRZ** in toluene at 77 K.


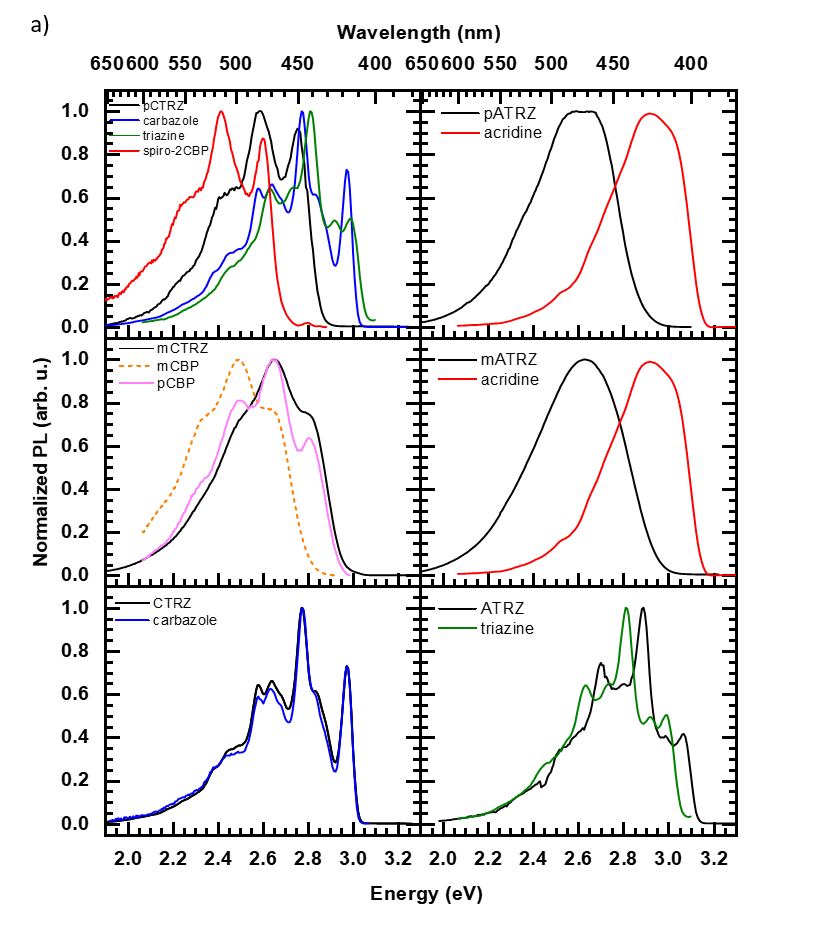


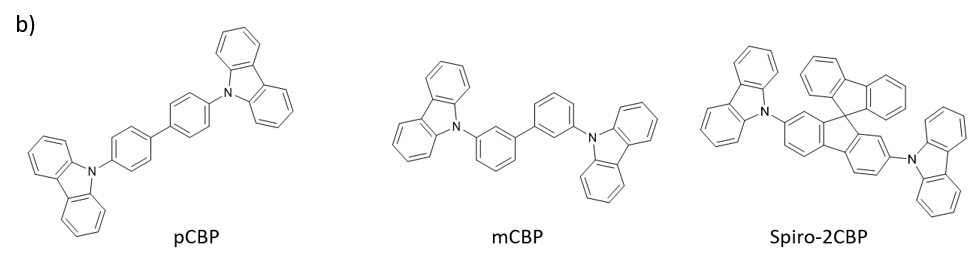

Fig S6. (a) Phosphorescence spectra **pCTRZ**, **mCTRZ**, **CTRZ**, **pATRZ**, **mATRZ** and **ATRZ** and corresponding modelling compounds in toluene at 77 K. All compounds except triazine were excited at 350 nm, and emission was detected after a 30ms delay with an integration of 15ms. Triazine solution was excited at 300 nm. (b) The chemical structures of pCBP, mCBP, Spiro-2CBP. The structures of carbazole, triazine and acridine are given in Fig. S1. (c) Comparison of the phosphorescence of the diphenylacridine, carbazole and diphenyltriazine units in toluene at 77 K. (d) comparison of the phosphorescence of of **Spiro-2CBP**, **CBP** and **mCBP** and their phosphorescence in hexane.

|  |  |
| --- | --- |

Figure S7. Steady-state emission in toluene at 77 K for carbazole (a) and acridine (b) series.

| (a) | (b) |
| --- | --- |

Figure S8. Decay of prompt fluorescence for **CTRZ** and **ATRZ** series in toluene solution at 300 K.

Concentration of the solution 0.05 mg/ml. Wavelength of excitation is 355 nm.

Figure S9. Phosphorescence (green line) and fluorescence (blue line) at 77K in toluene. The arrows indicate the energy difference between the two spectra.

Figure S10. Alternative representation of the NTOs of ATRZ in relaxed triplet T_1_ structure with the corresponding weight ν, calculated at the M06-2X/6-31+G(d) level. Top image represent the electron density e and bottom image the hole density h.

Table S1

Emission energies (in eV) in optimized S_1_ and T_1_ geometries calculated, both, at the M06-2X/6-31+G(d) and at the CAM-B3LYP/6-31+G(d) level. For comparison, the experimental values are also given for molecules in toluene at 77 K, as derived below.

|  | S_1_ ^(a)^  exp.  (eV) | T_1_^(b)^  exp.  (eV) | ΔE_ST_, exp. (eV) | S_1_  (M06-2X) | T_1_  (M06-2X) | ΔE_ST_ (eV) | S_1_  (CAM-B3LYP) | T_1_  (CAM-B3LYP) | ΔE_ST_ (eV) |
| --- | --- | --- | --- | --- | --- | --- | --- | --- | --- |
| **pCTRZ** | 3.22 | 2.76 | 0.46 | 3.48 | 2.69 | 0.79 | 3.61 | 2.57 | 1.04 |
| **mCTRZ** | 3.21 | 2.82 | 0.39 | 3.44 | 2.82 | 0.62 | 3.54 | 2.64 | 0.9 |
| **CTRZ** | 3.21 | 2.97 | 0.24 | 3.18 | 3.00 | 0.18 | 3.26 | 2.77 | 0.49 |
| **pATRZ** | 2.92 | 2.67 | 0.25 | 3.06 | 2.60 | 0.46 | 3.18 | 2.50 | 0.68 |
| **mATRZ** | 2.94 | 2.72 | 0.22 | 3.02 | 2.92 | 0.10 | 3.15 | 2.79 | 0.36 |
| **ATRZ** | 3.25 | 3.07 | 0.18 | 3.33 | 3.03 | 0.30 | 2.74 | 2.83 | -0.09 |

1. The energy of the singlet state was determined by fitting the spectral lineshape of the CT absorption and fluorescence, ^b)^ The energy of the triplet state was taken from the 0-0 the position of the phosphorescence at 77K.

Table S2

Comparison of the Singlet-Triplet gaps ΔE_ST_, obtained (1) as in Table 2 and Figure 4, and obtained (2) as in Figure S9.

|  | ΔE_ST_, exp.1 (eV) | ΔE_ST_, exp.2 (eV) |
| --- | --- | --- |
| **pCTRZ** | 0.46 | 0.43 |
| **mCTRZ** | 0.39 | 0.33 |
| **CTRZ** | 0.24 | 0 |
| **pATRZ** | 0.25 | 0.10 |
| **mATRZ** | 0.22 | 0.06 |
| **ATRZ** | 0.18 | 0 |
